# Multilingual Video Education for Hospitalized Patients With Myocardial Infarction (EDUCATE-MI): Single-Arm Implementation Study

**DOI:** 10.2196/82817

**Published:** 2026-03-26

**Authors:** Aileen Zeng, Edel O’Hagan, Sul Ki Kim, Simone Marschner, Mitchell Sarkies, Marina Wassif, Meng Ji, Julie Ayre, Daniel McIntyre, Clara K Chow, Aravinda Thiagalingam, Liliana Laranjo

**Affiliations:** 1 Westmead Applied Research Centre, Sydney Medical School, The University of Sydney, Level 5, Block K, Westmead Hospital, Hawkesbury Road, Westmead, 2145, Australia, +61 2 8890 3125, +61 2 8890 1960; 2 Department of Cardiology, Westmead Hospital, Westmead, Australia; 3 Westmead Institute for Medical Research, University of Sydney, Westmead, Australia; 4 School of Health Sciences, Faculty of Medicine and Health, University of Sydney, Sydney, Australia; 5 Sydney Health Partners, Implementation Science Academy, University of Sydney, Sydney, Australia; 6 School of Languages and Cultures, University of Sydney, Sydney, Australia; 7 Sydney Health Literacy Lab, School of Public Health, Faculty of Medicine and Health, The University of Sydney, Sydney, Australia

**Keywords:** myocardial infarction, health education, diversity, equity, inclusion, DEI, multilingual video

## Abstract

**Background:**

Clinical guidelines recommend the early initiation of secondary prevention strategies prior to hospital discharge for patients with myocardial infarction (MI) to reduce morbidity and mortality, but implementation is resource-intensive. Multilingual videos can deliver information in diverse preferred languages and literacy levels, but their impact on MI knowledge among hospitalized patients remains unclear.

**Objective:**

This study aims to assess whether the delivery of a multilingual educational video to hospitalized patients with MI can improve patient MI knowledge before hospital discharge.

**Methods:**

We conducted a single-arm pre-post study with embedded formative implementation evaluation from December 2023 to October 2024 in a tertiary hospital. The intervention was a video on post-MI management, available in English, Arabic, Hindi, and Mandarin (with Simplified Chinese subtitles). The intervention was delivered via a tablet provided by the research assistant. The primary outcome was the change in patient knowledge of MI, measured by comparing the mean number of correct responses before and after the intervention using a 2-tailed paired *t* test. We assessed early-stage implementation using 2 prespecified elements from the Proctor implementation outcomes framework: acceptability and fidelity of the video delivery. We performed content analysis on the notes taken from participants’ feedback to improve the video.

**Results:**

We recruited 129 participants (mean age of 59.4, SD 12.6 years) for this study. English was the preferred language (n=96, 74.4%) and Hindi was the predominant non-English language (n=17, 13.2%). Of the 129 participants enrolled, 128 completed follow-up immediately postintervention (1 lost interest). The average number of correct responses out of 10 was 5.4 (SD 2.7) at baseline and 7.2 (SD 2.5) postintervention (mean difference=1.9, 95% CI 1.6-2.2; *P*<.001; Cohen *d_rm_* for paired change=0.72). The educational video was well-accepted, with 83.6% (107/128) of participants finding it easy to understand, 74.2% (95/128) engaging, and 87.5% (112/128) useful. Participants’ feedback for improvement highlighted content complexity and a preference for conversational language and dialects. Fidelity of the intervention was subjectively assessed as reasonably achieved, given that the core components of the intervention (ie, animations and educational content conveyed through the audio and subtitles) were delivered as intended. Fidelity of the implementation strategy was similarly assessed as reasonably achieved because there were no technology issues preventing delivery of the intervention as intended, through video display from a weblink embedded in REDCap, using a tablet with internet connection.

**Conclusions:**

A short educational video may improve patient knowledge of MI before discharge. Further scaled research is needed to evaluate the effectiveness and implementation of this intervention in additional languages and diverse populations. This study highlights the need for culturally and linguistically tailored resources in clinical settings, informing future research and policy on inclusive patient education.

## Introduction

Cardiovascular disease (CVD), a group of diseases affecting the heart and blood vessels, remains the leading cause of death worldwide [1]. Most CVD mortality is due to atherosclerotic causes, including acute myocardial infarction (MI) secondary to plaque rupture (type 1 MI), accounting for 38% to 44% of all CVD-related deaths worldwide [2]. After the acute phase, the risk of death from another cardiac event remains high [3].

International guidelines recommend the early initiation of secondary prevention strategies prior to hospital discharge to reduce the risk of morbidity and mortality [4], but there are challenges in implementation. A key component of secondary prevention is adherence to various medications, including long-term aspirin and dual antiplatelet therapy [5]. However, adherence to these medications remains suboptimal [6] due to barriers including psychological factors and the complexity of the drug regimen [7]. Patient education is key to addressing MI knowledge gaps and suboptimal medication adherence, but inpatient education delivery relies on time-pressured frontline clinicians and may fail to accommodate the needs of diverse populations [8].

Educational videos can help deliver evidence-based health information to diverse populations and accommodate different language preferences and literacy levels, including people with limited reading ability or those who are illiterate [9,10]. A systematic review (59 experimental studies, n=9789) of video-based educational interventions reported improved knowledge in 75 % (30/40) of the assessed outcomes in people with chronic diseases [9]. Previous studies evaluating inpatient video interventions for patients post-MI [11,12] have shown improvements in patient knowledge, but the videos in these studies were delivered in 1 predominant language. To date, no studies have evaluated the impact of multilingual educational videos delivered during admission on patient knowledge post-MI. Assessing knowledge during hospitalization and before discharge is important as distress in the acute period of an MI can interfere with understanding and recalling clinical information [13].

The aim of this study was to assess whether the delivery of a multilingual educational video to hospitalized patients with MI can improve their patient knowledge of MI before hospital discharge.

## Methods

### Study Design

We conducted a single-arm pre-post study with embedded formative implementation evaluation in a tertiary teaching hospital in Sydney, Australia. The protocol is available on Open Science Framework (registered on April 12, 2024) [14]. Reporting of this study follows the StaRI (Standards for Reporting Implementation Studies; Checklist 1), TIDieR (Template for Intervention Description and Replication; Checklist 2) guidelines, and TREND (Transparent Reporting of Evaluations with Non-Randomized Designs; Checklist 3) [15-17].

### Patient and Public Involvement

Members of the public were not involved in the design of the study or the interpretation of the findings. There are plans to disseminate the results of the research to study participants and the community via our institute’s monthly newsletter.

### Participants and Setting

Patients were invited to participate if they were aged 18 years or older, had been admitted for inpatient services at Westmead Hospital for a type 1 MI [18], had undergone coronary angiography, and understood 1 of the 4 available languages: English, Arabic, Hindi, or Mandarin (with Simplified Chinese subtitles; Multimedia Appendix 1).

Participants who were unable to consent to the study in 1 of 4 four languages or who were unable to complete the video due to cognitive or visual impairments were excluded.

### Intervention and Implementation Strategy

The intervention was a single video with subtitles, approximately 5 minutes long, with voiceovers performed by native speakers in English, Hindi, Arabic, and Mandarin (Simplified Chinese subtitles) to ensure linguistic appropriateness. The video included different animations and focused on explaining the disease process leading to a type 1 MI (ie, acute coronary atherothrombosis) and the importance of different medications post-MI. The video was developed by a consultant cardiologist and a cardiology advanced trainee based on the latest clinical guidelines, using whiteboard animation software, with input from a multidisciplinary team. The development and description of the video is described following the TIDieR checklist in [16].

The implementation strategy was the delivery of the intervention via a tablet with an internet connection, provided by the research assistant. The video was embedded in REDCap (Research Electronic Data Capture; Vanderbilt University) surveys (accessed via a weblink) and hosted online.

### Recruitment and Data Collection

Eligible participants were identified through discussion with their care team and approached at the patient’s bedside. Participants were informed that they could withdraw at any time. We did not approach participants who were clinically unstable, as indicated by the ward clinicians. A research assistant conducted the consent process and stayed with the patient while they watched the video. Patients were encouraged to provide feedback on the video during and after visualization; the research assistant took notes of patients’ comments and feedback. At baseline, sociodemographic data and MI knowledge were collected. MI knowledge was assessed immediately postintervention and at a 1-month follow-up (the latter being optional, for participants who opted to provide an email address for this purpose), as well as data on the acceptability of the video. Sociodemographic data, MI knowledge, and acceptability data were collected via self-reported electronic questionnaires hosted on the REDCap platform.

### Study Outcomes

The prespecified primary outcome was prospectively defined as the average number of correct responses on the MI knowledge questionnaire immediately after the intervention, compared to baseline. Given the lack of validated questionnaires assessing MI knowledge [19], one of the investigators (AT) developed the MI knowledge questionnaire with contributions from other clinicians. The tool is a 10-item multiple-choice (5 options with 1 correct answer per question) questionnaire that assesses general MI knowledge, with questions on the causes of MI and post-MI medications (with the total number of correct responses ranging from 0=“no correct answers” to 10=“ all answers to the 10 questions were correct”; Multimedia Appendix 1).

A prespecified secondary outcome was the average number of correct responses in the MI knowledge questionnaire at 1 month. Exploratory post hoc outcomes included the proportion of participants who improved their total number of correct responses from baseline to postintervention, the proportion meeting the knowledge target (defined as 7 or more correct responses out of 10) or medication knowledge target (defined as correctly answering all 7 medication questions), and the proportion of correct responses for each of the 10 individual questions postintervention compared to baseline.

To assess early-stage implementation, we evaluated two prespecified elements from the Proctor implementation outcomes framework [20]: (1) acceptability of the intervention and (2) fidelity of the intervention and implementation strategy (ie, delivery of the intervention via a tablet with an internet connection). Acceptability of the intervention was assessed through content analysis of participants’ feedback (described in the “Data Analysis” section) and via a questionnaire asking participants to rate 3 different statements on a 5-point scale from strongly disagree to strongly agree (“The information delivered in the video was easy enough to understand,” “I found the video engaging,” and “I found the information useful”) and asking whether they would be interested in receiving similar videos in the future (yes or no).

Fidelity of the intervention was subjectively assessed by the investigators based on whether the core components of the intervention (ie, video animations and educational content conveyed through the audio and subtitles) were delivered as intended across the 4 different language versions. For this assessment, perspectives from the translators involved in the study were sought regarding the extent to which the translated content matched the English version. Fidelity of the implementation strategy was subjectively assessed by the investigators based on deviations from the intended mode of delivery (ie, video display from a weblink embedded in REDCap, using a tablet with internet connection).

### Data Analysis

Data analysis followed a statistical analysis plan developed a priori (Open Science Framework) [14]. A sample size of 119 participants was estimated to provide 90% power to detect a moderate effect size (d=0.3) in knowledge difference pre-post, considering a 2-sided type 1 error of 0.05.

We expressed descriptive data as proportions and means with SD. The prespecified primary outcome was analyzed using a 2-tailed paired *t* test, measuring the mean difference in patient knowledge before and after the intervention, reported with 95% CIs, and Cohen *d_rm_* for paired change (Multimedia Appendix 1). An exploratory McNemar test was used to assess changes in the proportion of participants who met knowledge targets before and immediately postintervention, as well as the proportion of correct responses for each of the 10 individual questions postintervention compared to baseline. A prespecified 2-tailed paired *t* test was used to measure the mean difference in patient knowledge before and 1 month postintervention. To test the homogeneity of the treatment effect across subgroups, we used ANCOVA (postadjusted for baseline) to assess the change in the average number of correct responses by age (≤65 y and >65 y), sex (male and female), language (English and non-English), and education (prior to secondary education completion and postsecondary education; Multimedia Appendix 1). We report the proportion of participants who improved their total number of correct responses from baseline to postintervention and implementation measures of acceptability descriptively immediately postintervention and at 1-month follow-up.

Analyses were conducted using R statistical software (version 4.4.0; R Project for Statistical Computing). *P* value was 2-sided, and statistical significance was set at *P*=.05. Data were analyzed from October 2024 to November 2024. Participants with missing data at follow-up were excluded from the analysis. Two researchers performed content analysis on the notes taken from participants’ feedback. We were interested in understanding the participants’ perspectives on the content of the video in the different languages and their suggestions for improving the videos. Themes were reviewed and discussed with 2 other investigators to clarify, explore, and refine interpretations.

### Ethical Considerations

Ethical approval (2021_ETH00983_v3) was obtained from the Western Sydney Local Health District Human Research Ethics Committee. Participant information and consent forms were available in English, Arabic, Hindi, and Mandarin (Simplified Chinese subtitles). Informed consent was obtained in the participants’ preferred language. All documents and content in the intervention were translated by the multicultural unit in the Western Sydney Local Health District by a qualified health interpreter. Data were collected and stored on secure servers accessible only to approved study personnel. All data were deidentified for data analysis and publication. No compensation was offered to the participants.

## Results

**Figure 1.**
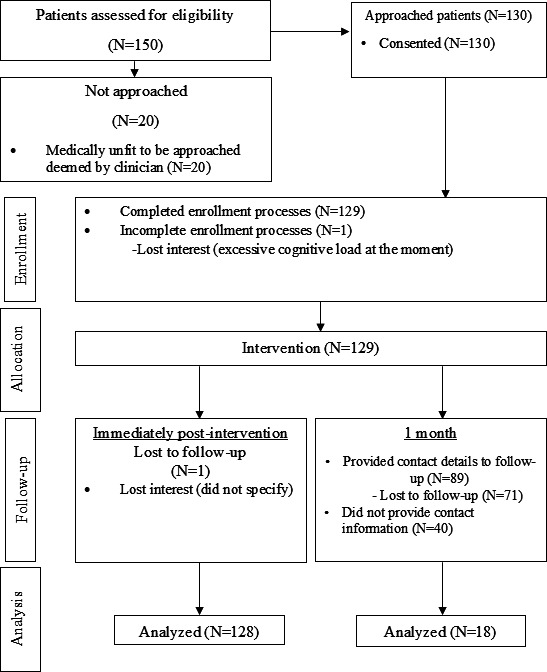
Flow diagram of participants in the single-arm pre-post study.

Between December 2023 and October 2024, a total of 150 patients were assessed for eligibility. Of these, 130 were approached (20 were deemed by a clinician as not medically fit to be approached for the study), and 129 were enrolled (Figure 1). Of the 129 participants enrolled, 128 completed follow-up immediately postintervention (1 lost interest). Of the 89 participants who provided their email address for the 1-month survey, 18 responded (Figure 1). The baseline characteristics of participants are summarized in Table 1. The mean participant age was 59.4 (SD 12.6) years and 20.2% (26/129) were female. In our sample, 24.8% (32/129) were South Asian and 76% (98/129) completed secondary education or above. The majority of our participants listed English as their preferred language (96/129, 74.4%), followed by Hindi (17/129, 13.2%), Arabic (10/129, 7.8%), and Mandarin (with Simplified Chinese subtitles; 6/129, 4.7%).

**Table 1.** Baseline sociodemographic and clinical characteristics of participants (N=129).

Characteristics^a^	Value
Sex, n (%)	
Female	26 (20.2)
Age (y; missing=3), mean (SD)	59.4 (12.6)
Ethnicity, n (%)	
Aboriginal or Torres Strait Islander	3 (2.3)
Australian or New Zealander	25 (19.4)
Polynesian	1 (0.8)
European	29 (22.5)
American (North, Central, and South)	1 (0.8)
South Asian (Bangladesh, India, Nepal, Pakistan, and Sri Lanka)	32 (24.8)
East Asian (China, Japan, and Taiwan)	8 (6.2)
South-East Asian (Vietnam, Cambodia, Laos, Burma, Malaysia, Singapore, Philippines, Thailand, Indonesia, and East Timor)	8 (6.2)
Middle East and North African	19 (14.7)
Sub-Saharan Africa	1 (0.8)
Pacific Islander	2 (1.6)
Other	0 (0)
Preferred languages, n (%)	
English	96 (74.4)
Arabic	10 (7.8)
Hindi	17 (13.2)
Mandarin (with Simplified Chinese subtitles)	6 (4.7)
Education level, n (%)	
Never attended school	1 (0.8)
Primary	7 (5.4)
Secondary school without completion certificate	23 (17.8)
Secondary school graduate	25 (19.4)
Technical or vocational qualifications	10 (7.8)
University undergraduate	47 (36.4)
University postgraduate	16 (12.4)
Risk factors, n (%)	
Diabetes	48 (37.2)
Hypertension	74 (57.4)
High cholesterol	66 (51.2)
Smoker (or recently quit <12 mo)	47 (36.4)

a All data are self-reported.

**Figure 2.**
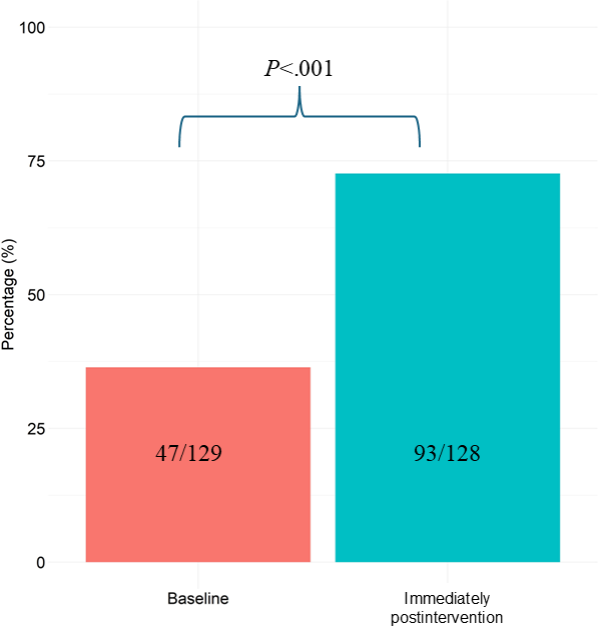
Bar plot illustrating the proportion of participants who met the knowledge target (defined as 7 or more correct responses out of 10) before and immediately after the video intervention. Changes in the proportion of participants were assessed using a McNemar test.

In the primary outcome analysis, the average number of correct responses was 5.4 (SD 2.7) at baseline and 7.2 (SD 2.5) postintervention (mean difference=1.9, 95% CI 1.6-2.2; *P*<.001; Cohen d_rm_ for paired change=0.72; Table 2). At 1 month postintervention, there were 18 completed responses (Multimedia Appendix 1). Overall, 72.7% (93/128) of the participants showed higher scores in their total number of correct responses postintervention compared to baseline (Multimedia Appendix 1). The proportion of participants who met the MI knowledge target postintervention increased from 47/129 (36.4%) to 93/128 (72.7%; *P*<.001; Figure 2). For each of the individual questions, the proportion of correct responses increased from baseline to postintervention, except for 1 question (question 7; Multimedia Appendix 1).

Regarding the acceptability of the intervention, most participants reported that they agreed or strongly agreed that the information delivered in the video was easy to understand (107/128, 83.6%), engaging (95/128, 74.2%), and useful (112/128, 87.5%); additionally 84.4% (108/128) reported they would like to receive similar videos in the future (Multimedia Appendix 1).

**Table 2.** Mean difference in myocardial infarction (MI) knowledge before and immediately after the multilingual video intervention in hospitalized patients with MI prior to discharge (n=128).

	Baseline, n=129	Immediately postintervention, n=128	Mean difference, n=128^a^ (95% CI)	*P* value	Cohen *d_rm_*^b^
Mean number of correct responses^c^, mean (SD)	5.4 (2.7)	7.2 (2.5)	1.9 (1.6-2.2)	<.001	0.72

a Analyzed using 2-tailed paired *t* test.

b Cohen *d*_rm_ for paired change - adjusted *d*_z_ estimating the “standard“ between-subjects *d* by a factor of $\sqrt{2\left(1-r\right)}$, where *r* is the Pearson correlation between the paired measures [21] (Supplement F in Multimedia Appendix 1).

c Correct responses range from 0 to 10.

Nine participants (5 Hindi-speaking, 2 Mandarin-speaking, and 2 Arabic-speaking) provided qualitative feedback on the videos. Content analysis of the feedback revealed two main themes: (1) complexity of the content and medical jargon, and (2) preference for conversational language and dialects. Regarding the complexity of the content and medical jargon, participants indicated they had difficulty understanding some terms in the videos, expressing a preference for lay language. Examples of quotes from participants grouped in this theme include:

*The video will be too difficult to understand (for people not) familiar with the terminology.*

[Study ID 125; 70‐75 years; preferred language: Hindi; educational level: university undergraduate]

*High-order Hindi was used—needs to have less jargon.*

[Study ID 30; 66‐70 years; preferred language: Hindi; educational level: university postgraduate]

Regarding the preference for conversational language and dialects, participants with Hindi as their preferred language reported that a macaronic-hybrid language was commonly used to communicate and would be preferred for the video. Participants with Arabic as their preferred language mentioned that they would prefer the video to be in their specific dialect. Examples of quotes are presented as follows:

*Mix of Hindi and English would have been better—conversational Hindi.*

[Study ID 30; 66-70 years; preferred language: Hindi; educational level: university postgraduate]

*Dialect is like Egyptian… parents would understand Nahw… (I) am bilingual—so can’t read or write.*

[Study ID 108; 40-45 years; preferred language: Arabic; educational level: university undergraduate]

Fidelity of the intervention was subjectively assessed as reasonably achieved, given that the core components of the intervention (ie, animations and educational content conveyed through the audio and subtitles) were delivered as intended. The video animations were the same across the 4 translated versions of the educational video; only the timing between different animations changed to accommodate the audio. The educational content conveyed through the audio and subtitles was also delivered as intended, with minor differences in language between the different translations. Fidelity of the implementation strategy was similarly assessed as reasonably achieved because there were no technology issues preventing delivery of the intervention as intended through video display from the weblink embedded in REDCap, using a tablet with internet connection.

## Discussion

### Principal Findings

In this single-arm pre-post study with embedded formative implementation evaluation of 128 inpatients admitted for an MI, we found that a short multilingual educational video may improve patient knowledge of MI before discharge. The videos were deemed acceptable, with most participants finding them easy to understand, engaging, and useful. The core components of the intervention were delivered as intended, as the videos across the 4 languages comprised the same animations and educational content in both audio and subtitles, with only slight timing differences due to language nuances between translations. There were no deviations from the implementation strategy, with all participants watching the video through a REDCap weblink on a tablet with internet connection, as intended. Our study demonstrates that it is possible to deliver videos in different languages—English, Arabic, Hindi, and Mandarin (with Simplified Chinese subtitles)—to inpatients in a linguistically diverse context at a tertiary hospital, with modest improvement in MI knowledge before discharge.

### Comparisons With Prior Work

Results from our study suggest that a single multilingual video intervention may improve patient MI knowledge in the inpatient setting. Our results are consistent with other studies evaluating inpatient video interventions for patients post-MI [11,12], although ours was the only one delivered in more than 1 language. One of these studies, a randomized controlled trial (RCT) of 68 inpatients, reported a moderate within-subject change in the intervention group (15-min video) at 3-month follow-up in the same order of magnitude that we found in our study [11]. A 2-arm quasi-experimental study (N=25) reported a 50% larger within-subject change in the intervention group at 7-day follow-up [12]. These medium to large within-subject changes also appear to persist when video interventions are delivered in a postdischarge cardiac rehabilitation setting. A pre-post study of a culturally adapted secondary prevention video education program (Simplified Chinese) for patients post-MI reported that one-quarter of patients found the information overwhelming, despite a medium within-subject change in knowledge similar in magnitude to our findings [22]. Given the competing demands during MI recovery, even the modest knowledge gains noted may be clinically meaningful and are consistent with previous findings. It remains unclear how different health literacy strategies applied to multilingual videos can influence knowledge in different populations, which should be explored in future research [23].

The mean postintervention score was 7/10, even after participants had just viewed the video, highlighting the persistent challenges of meeting patients’ educational and health literacy needs. Despite efforts to adhere to readability recommendations (ie, grade 8 or below), our findings indicate additional attention is required to produce simpler content with fewer unfamiliar terms. These findings were echoed in our feedback discussions and may reflect broader barriers to comprehending health resources, such as high readability levels (ie, above the recommended grade 8 level) and the use of medical jargon [24]. Reducing readability levels prior to translation into non-English languages is important, as content complexity may be compounded during translation [24,25]. In addition, the literal translation of certain terms may not accurately convey their intended meaning, and the use of culturally equivalent terms may be preferable [25,26]. Hence, cultural adaptation is key to adequately considering the cultural aspects that may not be captured through linguistic translation alone, such as cultural equivalence, cultural appropriateness, similarity of interpretability, and item relevance [25,27].

While knowledge scores improved postintervention and the video in our study was well-accepted, further gains may be achieved with the involvement of community members in the co-design and cultural adaptation of intervention content [24,28]. Notwithstanding the importance of addressing language barriers in improving health outcomes and quality of care [29], future studies aiming to develop equitable health education interventions should also consider the nuances of social context and how language intersects with race, migration status, religion, socioeconomic position, and other social determinants of health [30-32].

### Strengths and Limitations

Strengths of this study include delivering the intervention to a diverse, multicultural population (n=114, 88% non-Caucasian) in an inpatient setting. We translated the intervention into 4 different languages and adapted the medical jargon in the translations. However, there were challenges in translating medical language, and the readability of our English-language content was high, at grade 10, instead of the desired grade 8. These limitations are common in medical content, where the inclusion of specific, complex, condition-specific terminology (eg, MI) is often unavoidable and increases overall readability scores [33].

This study should be interpreted in the context of its single-arm pre-post design, which limits the evaluation of causality. Our English educational content did not meet the recommended grade 8 readability levels and was at a grade 10 level due to the inclusion of unavoidable medical terms, which were otherwise explained in the transcript. Translations were reviewed by native speakers, but more comprehensive patient involvement in co-design and cultural adaptation will be key for future iterations, based on participant feedback. Only 34 out of 129 participants received the intervention in a language other than English, which reflects the known challenges of recruiting participants from diverse backgrounds [34,35]. Furthermore, our content analysis of patient feedback was based on researcher notes rather than verbatim transcripts of recorded feedback, limiting opportunities for in-depth analysis of participants’ perspectives of the intervention. Patient knowledge may have been influenced by short-term recall in the immediate postintervention period, and the 1-month results should be interpreted with caution due to low response rates and the risk of survivor bias. Given the absence of validated patient questionnaires to assess MI knowledge, the MI knowledge questionnaire was created by clinicians in the study and was not psychometrically assessed. It may not measure actionable MI knowledge, which is key for patient empowerment and behavior change. Finally, we did not assess medication adherence, secondary prevention behaviors, health literacy, or language proficiency, which could have aided in the interpretation of the results.

Multilingual videos have the potential to reduce language barriers in the delivery of inpatient education for patients with MI prior to discharge, particularly in health care services that serve multicultural communities. We demonstrated that it is possible to improve knowledge in the short term and implement educational videos within a 5-minute timeframe for hospitalized patients with MI in a multicultural context. This offers a scalable and pragmatic strategy that may ease demands on health care staff prior to discharge. Patients report a considerable treatment burden in the first year following an MI, particularly those with lower health literacy [36], which may contribute to suboptimal medication adherence. Providing education prior to discharge may help alleviate this burden and serve as a primer for adherence to secondary prevention care. In the future, large language models and other generative artificial intelligence tools could complement video-based education by providing interactive, personalized support [37] in a patient’s preferred language and at an appropriate readability level [38]. These tools could enhance health literacy and support equitable care for patients from diverse linguistic backgrounds. Future research, particularly larger RCTs with longer follow-up, should consider strategies to better engage participants with non-English language preferences, to enhance inclusivity, and evaluate the effectiveness of multilingual patient education interventions in different language groups.

### Conclusions

A short educational video delivered during inpatient hospital admission for acute MI may improve patient knowledge before discharge. Our study demonstrates that it is possible to deliver videos in different languages—English, Arabic, Hindi, and Mandarin (with Simplified Chinese subtitles)—to patients in a multicultural context at a tertiary hospital. Future interventions should be culturally adapted and co-designed with patients. Further RCTs are needed to evaluate the long-term impact of this intervention on MI knowledge and secondary prevention behaviors across different settings.

## Supplementary material

10.2196/82817 Multimedia Appendix 1 Supplementary material (Setting Details, Protocol Deviations, MI Knowledge Questionnaire, and Detailed Analyses).

10.2196/82817 Checklist 1 StaRI checklist.

10.2196/82817 Checklist 2 TiDier checklist.

10.2196/82817 Checklist 3 TREND checklist.

10.2196/82817 Checklist 4 iCHECK-DH checklist.

## Abbreviations

CVD: cardiovascular disease
MI: myocardial infarction
RCT: randomized controlled trial
REDCap: Research Electronic Data Capture
StaRI: Standards for Reporting Implementation Studies
TIDieR: Template for Intervention Description and Replication
TREND: Transparent Reporting of Evaluations with Non-Randomized Designs

## References

[1] Murray CJL (2022). The Global Burden of Disease Study at 30 years. Nat Med.

[2] Timmis A, Vardas P, Townsend N (2022). European Society of Cardiology: cardiovascular disease statistics 2021. Eur Heart J.

[3] Nair R, Johnson M, Kravitz K (2021). Characteristics and outcomes of early recurrent myocardial infarction after acute myocardial infarction. J Am Heart Assoc.

[4] Byrne RA, Rossello X, Coughlan JJ (2023). ESC guidelines for the management of acute coronary syndromes: developed by the task force on the management of acute coronary syndromes of the European Society of Cardiology (ESC). Eur Heart J.

[5] Laranjo L, Lanas F, Sun MC (2024). World Heart Federation roadmap for secondary prevention of cardiovascular disease: 2023 update. Glob Heart.

[6] Naderi SH, Bestwick JP, Wald DS (2012). Adherence to drugs that prevent cardiovascular disease: meta-analysis on 376,162 patients. Am J Med.

[7] Brown MT, Bussell JK (2011). Medication adherence: WHO cares?. Mayo Clin Proc.

[8] Geerligs L, Rankin NM, Shepherd HL, Butow P (2018). Hospital-based interventions: a systematic review of staff-reported barriers and facilitators to implementation processes. Implement Sci.

[9] Deshpande N, Wu M, Kelly C (2023). Video-based educational interventions for patients with chronic illnesses: systematic review. J Med Internet Res.

[10] Sarkies MN, Maloney S, Symmons M, Haines TP (2019). Video strategies improved health professional knowledge across different contexts: a helix counterbalanced randomized controlled study. J Clin Epidemiol.

[11] Bruggmann C, Adjedj J, Sardy S, Muller O, Voirol P, Sadeghipour F (2021). Effects of the interactive web-based video “Mon Coeur, Mon BASIC” on drug adherence of patients with myocardial infarction: randomized controlled trial. J Med Internet Res.

[12] Winiger AM, Shue-McGuffin K, Moore-Gibbs A, Jordan K, Blanchard A (2021). Implementation of an Ask Me 3 ® education video to improve outcomes in post-myocardial infarction patients. Am J Prev Cardiol.

[13] Whitehead DL, Perkins-Porras L, Strike PC, Steptoe A (2006). Post-traumatic stress disorder in patients with cardiac disease: predicting vulnerability from emotional responses during admission for acute coronary syndromes. Heart.

[14] Clinician-created multimedia and multicultural cardiovascular m-health education—EDUCATE-MI. Open Science Framework.

[15] Pinnock H, Barwick M, Carpenter CR (2017). Standards for Reporting Implementation Studies (StaRI) statement. BMJ.

[16] Hoffmann TC, Glasziou PP, Boutron I (2014). Better reporting of interventions: template for intervention description and replication (TIDieR) checklist and guide. BMJ.

[17] Des Jarlais DC, Lyles C, Crepaz N, TREND Group (2004). Improving the reporting quality of nonrandomized evaluations of behavioral and public health interventions: the TREND statement. Am J Public Health.

[18] Thygesen K, Alpert JS, Jaffe AS (2018). Fourth universal definition of myocardial infarction (2018). Circulation.

[19] Fajardo MA, Yung C, Cornell S (2025). Quality of available cardiovascular disease knowledge tools: a systematic review. Glob Heart.

[20] Proctor E, Silmere H, Raghavan R (2011). Outcomes for implementation research: conceptual distinctions, measurement challenges, and research agenda. Adm Policy Ment Health.

[21] Ben-Shachar M, Lüdecke D, Makowski D (2020). Effectsize: estimation of effect size indices and standardized parameters. J Open Source Softw.

[22] Shi W, Zhang L, Ghisi GLM, Panaretto L, Oh P, Gallagher R (2024). Evaluation of a digital patient education programme for Chinese immigrants after a heart attack. Eur J Cardiovasc Nurs.

[23] The Patient Education Materials Assessment Tool (PEMAT) and user’s guide. Agency for Healthcare Research and Quality.

[24] Shan Y, Ji M, Dong Z, Xing Z, Wang D, Cao X (2023). The Chinese version of the Patient Education Materials Assessment Tool for printable materials: translation, adaptation, and validation study. J Med Internet Res.

[25] Shan Y, Xing Z, Dong Z, Ji M, Wang D, Cao X (2023). Translating and adapting the DISCERN instrument into a simplified Chinese version and validating its reliability: development and usability study. J Med Internet Res.

[26] Thakkar J, Karthikeyan G, Purohit G (2016). Development of macaronic Hindi-English “Hinglish” text message content for a coronary heart disease secondary prevention programme. Heart Asia.

[27] Mac O, Ayre J, McCaffery K, Boroumand F, Bell K, Muscat DM (2025). The readability study: a randomised trial of health information written at different grade reading levels. J Gen Intern Med.

[28] Henderson S, Kendall E, See L (2011). The effectiveness of culturally appropriate interventions to manage or prevent chronic disease in culturally and linguistically diverse communities: a systematic literature review. Health Soc Care Community.

[29] Diamond L, Izquierdo K, Canfield D, Matsoukas K, Gany F (2019). A systematic review of the impact of patient-physician non-English language concordance on quality of care and outcomes. J Gen Intern Med.

[30] Reppas-Rindlisbacher C, Rawal S (2024). Understanding linguistic inequities in healthcare: moving from the technical to the social. BMJ Qual Saf.

[31] Abdi I, Tinessia A, Mahimbo A, Sheel M, Leask J (2025). Is it time to retire the label “CALD” in public health research and practice?. Med J Aust.

[32] Javed Z, Haisum Maqsood M, Yahya T (2022). Race, racism, and cardiovascular health: applying a social determinants of health framework to racial/ethnic disparities in cardiovascular disease. Circ Cardiovasc Qual Outcomes.

[33] Ayre J, Bonner C, Muscat DM (2023). Multiple automated health literacy assessments of written health information: development of the SHeLL (Sydney Health Literacy Lab) Health Literacy Editor v1. JMIR Form Res.

[34] Watson E, Gulline H, Jane SM, Woollett A, Ayton D (2025). Improving participation of culturally and linguistically diverse participants in clinical trials: an expert consultation. Trials.

[35] Knepper TC, McLeod HL (2018). When will clinical trials finally reflect diversity?. Nature.

[36] Qu W, Wang X, Zhang S (2025). Factors related to the treatment burden of patients with coronary heart disease: a cross-sectional study. Heart Lung.

[37] O’Hagan E, McIntyre D, Laranjo L (2023). The potential for a chat-based artificial intelligence model to facilitate educational messaging on hypertension. Hypertension.

[38] Ayre J, Mac O, McCaffery K (2024). New frontiers in health literacy: using ChatGPT to simplify health information for people in the community. J Gen Intern Med.

