# Supplementary material for: Multilingual Video Education for Hospitalized Patients With Myocardial Infarction (EDUCATE-MI): Single-Arm Implementation Study
Source: JMIR Cardio. 2026 Mar 26;10:e82817. doi: 10.2196/82817 (PMC13020905; doi:10.2196/82817)
Supplement: Multimedia Appendix 1 [file cardio-v10-e82817-s001.docx]

**Supplements**

[**Supplement D| eMethods 4: Details on the Setting and Deviations from the Protocol** 3](#_Toc223512908)

[**Supplement E| eMethods 5: MI Knowledge Questionnaire** 4](#_Toc223512909)

[**Supplement F| eMethod 6: Cohen’s d calculation for paired change of Primary Outcome** 6](#_Toc223512910)

[**Supplement G| Table S1: Subgroup Analysis** 6](#_Toc223512911)

[**Supplement H| Table S2-4: Analysis of 1-month post-intervention participant responses** 7](#_Toc223512912)

[**Supplement I| Table S5- 16: Post hoc Analysis** 9](#_Toc223512913)

[**Table S5. Proportion of participants who improved their total number of correct responses from baseline to post-intervention** 9](#_Toc223512914)

[**Table S6-S16: Exploratory Ten item level McNemar tests** 9](#_Toc223512915)

[**Table S16: Proportion of participants who met medication knowledge target (correctly answering all 7 medication questions)** **comparing pre-intervention to post-intervention baseline vs post-intervention** 14](#_Toc223512916)

[**Supplement J| Table S17. Acceptability measures immediately post-intervention and at 1 month follow-up** 14](#_Toc223512917)

## **Supplement D| eMethods 4: Details on the Setting and Deviations from the Protocol**

The coronary care unit at Westmead Hospital consists of 12 beds and provides comprehensive cardiac care to a diverse patient population, of which 47% are born overseas and 50% speak a language other than English. The median length of stay for a Type I MI at Westmead Hospital is 3 days.

The intervention (i.e. a video) was designed by investigators (AT and SK). In brief, after consultation with the latest acute coronary syndrome guidelines, investigators developed a transcript in English that addressed the fundamental concepts of MI pathophysiology, and management (particularly focussing on medications). The video’s sequence with animations were created with Video Scribe video animation software version 3.6 (Sparkol) at a cost ($AUD42 per month). A voiceover of the transcript was recorded by SK for the English language video, and we ensured the voiceover corresponded to the video sequence. Voiceovers for other languages were performed by trained health care services interpreters (Arabic and Mandarin) and a bilingual physician (Hindi). The Video Scribe software allowed the visual content timing to be modified to synchronise with the different timings of alternate languages.

Deviations from the protocol

Deviations from the protocol include the exploratory analysis of the following secondary outcomes: the proportion of participants who improved their total number of correct answers from baseline to post-intervention, the proportion meeting the knowledge target (defined as 7 or more correct responses out of 10), and the proportion of correct responses for each of the ten individual questions post-intervention compared to baseline.

## **Supplement E| eMethods 5: MI Knowledge Questionnaire**

The correct responses are bolded below:

| Question | Responses |
| --- | --- |
| Q1: A heart attack is most commonly caused by? | - blockage of a heart vein - **blockage of a heart artery** - damage to a heart valve - extreme stress or shock - I don't know |
| Q2. Blood thinning medications given after a myocardial infarction (heart attack) reduce blood clot by working on: | - **Platelets** - Red blood cells - White blood cells - Blood proteins - I don't know |
| Q3. Aspirin is used after myocardial infarction as a: | - For pain relief - To lower cholesterol - To lower blood pressure - **A blood thinner to stop blood clots** - I don't know |
| Q4. After a heart attack aspirin is an essential medication which should be taken: | - Short term for a month - **Long term until instructed to stop by your doctor** - As needed if you feel chest pain - Not at all in most cases - I don't know |
| Q5. Clopidogrel ('Plavix') and Ticagrelor ('Brilinta') are blood thinners used after a myocardial infarction which are given to: | - **Stop blood clot forming on the stent within the heart artery** - Reduce cholesterol or fat in the body - Lifelong - Not at all in most cases - I don't know |
| Q6. After a heart attack the most effective way of preventing future heart attacks is to: | - **Treatment with cholesterol lowering medications** - Regular angiograms to look at the heart arteries and see if another stent is needed - Regular blood tests of cholesterol levels - No special treatment needed because it is rare to get a second heart attack - I don't know |
| Q7. Narrowing of the coronary (heart) arteries is usually caused by: | - The arteries shrinking due to overuse - A build up of muscle in the heart artery wall - **A build up of fat in the heart artery wall** - Kinking of the heart arteries - I don't know |
| Q8. Cholesterol lowering medications are prescribed after a myocardial infarction: | - **Regardless of your cholesterol level** - If you have high levels of calcium in your blood - Only if your blood pressure is high - If you have high cholesterol levels - I don't know |
| Q9. Medications such as ACE inhibitors (ending in 'pril') or ARBs (ending in 'sartan') reduce the amount of work that the heart has to do by: | - **reducing the blood pressure** - increasing the heart rate - reducing fluid overload - improving lung function - I don't know |
| Q10. Beta blockers reduce stress on the heart by: | - increasing the blood pressure - **reducing the heart rate** - reducing fluid overload - improving lung function - I don't know |

Scoring Guide: Correct responses are bolded. Each correct response equates to 1 point. The maximum total score is 10 and the minimum is 0. Medication-specific questions correspond to Question 3,4,5,6,8,9, and 10.

## **Supplement F| eMethod 6: Cohen’s d calculation for paired change of Primary Outcome**

We calculated Cohen *d* for paired change of our primary outcome by using the function (repeated_measures_d) from the R package: effectsize.^4^ We standardized the mean difference for repeated measures by within-subject variance.

**Within-Subject Variance: *d_rm_ -*** Adjusted *d_z_*  estimating the "standard" between-subjects *d* by a factor of $\sqrt{2(1-r)}$​, where *r* is the Pearson correlation between the paired measures.^5^

Cohen *d_rm_* = 0.72

**References**

4. Ben-Shachar MS, Lüdecke D, Makowski D. effectsize: Estimation of effect size indices and standardized parameters. Journal of open source software. 2020 Dec 23;5(56):2815.

5. Cohen J. Statistical power analysis for the behavioral sciences. routledge; 2013 May 13.

## **Supplement G| Table S1: Subgroup Analysis**

**Table S1. Mean number of correct responses baseline vs post-intervention by age , sex, language, education**

|  |  | Mean number of correct responses | | |  |  |  |  |
| --- | --- | --- | --- | --- | --- | --- | --- | --- |
| Subgroup | | Baseline  (SD) | Immediately post Intervention  (SD) | Mean change (SD)# | Raw Mean difference between subgroups [95% CI] |  | Adjusted Mean difference between subgroups [95%CI]† | P-value |
| Age (N=126)*  ≤65 (N=83) | | 5.72 (2.54) | 7.70 (2.26) | 1.98 (1.91) | -0.21[-0.91, 0.49] |  | -0.65 [-1.28, -0.03] | 0.04 |
| >65 (N=43) | | 4.47 (2.68) | 6.23 (2.52) | 1.77 (1.88) |  |  |  |  |
| Language (N=128)^  English speaker  (N=95) | | 5.72 (2.32) | 7.51 (2.35) | 1.81 (1.80) | -0.28 [-1.03, 0.47] |  | 0.15 [-0.53, 0.83] | 0.66 |
| Non-English speaker  (N=33) | | 4.39 (3.36) | 6.48 (2.62) | 2.09 (2.13) |  |  |  |  |
| Sex (N=128)^  Female (N=25) | | 4.73 (3.29) | 6.68 (3.05) | 2.08 (1.98) | 0.25 [-0.58, 1.07] |  | -0.07 [-0.81, 0.67] | 0.86 |
| Male (N=103) | | 5.54 (2.49) | 7.38 (2.28) | 1.83 (1.87) |  |  |  |  |
| Education (N=128)^  Post Secondary (N=97) | | 5.81 (2.51) | 7.64 (2.19) | 1.86 (1.83) | -0.11 [-0.88, 0.65] |  | 0.50 [-0.20,1.21] | 0.16 |
| Prior to Secondary (N=31) | | 4.03 (2.80) | 6.00 (2.84) | 1.97 (2.09) |  |  |  |  |
| *Missing: 3 participants (1 participant missing age and follow-up; 2 participants missing age at baseline); ^Analysed 128 out of 129 enrolled participants due to 1 participant lost to follow up immediately post-intervention.; #Difference in mean number of correct responses at follow-up compared to baseline, per participant.; † ANCOVA: post adjusted for baseline. | | | | | | | | |

## **Supplement H| Table S2-4: Analysis of 1-month post-intervention participant responses**

**Table S2: Comparing the average number of correct responses baseline vs 1-month post-intervention**

|  | Baseline  N=18 | 1 month post intervention  N=18 | Mean Difference (SD)  N=18*^*  [95%CI] | p-value |
| --- | --- | --- | --- | --- |
| Mean number of correct responses* (SD) | 6.3 (2.4) | 8.8  (1.6) | 2.5  (1.7)  [95%CI 1.7, 3.3] | < 0.001 |
| *Correct responses are out of 10 |  |  |  |  |
| ^Analysed with paired t test |  |  |  |  |

At 1-month post-intervention, there were 18 completed responses. The average number of correct responses increased from 6.3 (2.4) at baseline to 8.8 (SD 1.6) at one month (mean difference = 2.5; 95% CI 1.7, 3.3 , p<.001).

**Table S3: Baseline Demographics of participants who responded at 1 month**

| **Characteristic*** | **N = 18** |
| --- | --- |
| **Sex, n(%)** |  |
| Female | 3/18 (16.7) |
| **Age,** Mean (SD) | 55.3 (11.0) |
| **Ethnicity, n(%)** |  |
| Aboriginal/Torres Strait Islander | 1 / 18 (5.6) |
| Australian/New Zealand | 7 / 18 (38.9) |
| Polynesian | 0 / 18 (0) |
| European | 0 / 18 (0) |
| American (North, Central and South) | 0 / 18 (0) |
| South Asian (Bangladesh, India, Nepal, Pakistan, Sri Lanka) | 7 / 18 (38.9) |
| East Asian (China, Japan, Taiwan) | 1 / 18 (5.6) |
| South-East Asian (Vietnam, Cambodia, Laos, Burma, Malaysia, Singapore, Philippines, Thailand, Indonesia, East Timor) | 1 / 18 (5.6) |
| Middle-East and North African | 1 / 18 (5.6) |
| Sub-Saharan Africa | 0/ 18 (0) |
| Pacific Islander | 0 / 18 (0) |
| Other | 0 / 18 (0) |
| **Preferred Languages, n(%)** |  |
| Arabic | 1 / 18 (5.6) |
| Mandarin (Simplified Chinese subtitles) | 1 / 18 (5.6) |
| English | 12 / 18 (66.7) |
| Hindi | 4 / 18 (22.2) |
| **Education level, n(%)** |  |
| Never attended school | 0 / 18 (0) |
| Primary | 0 / 18 (0) |
| Secondary school without completion certificate | 3 / 18 (16.7) |
| Secondary school graduate | 2 / 18 (11.1) |
| Technical or Vocational qualifications | 3/ 18 (16.7) |
| University undergraduate | 6 / 18 (33.3) |
| University postgraduate | 4 / 18 (22.2) |
| **Risk Factors, n(%)** |  |
| Diabetes | 6 / 18 (33.3) |
| Hypertension | 9 / 18 (50) |
| High Cholesterol | 8 / 18 (44.4) |
| Smoker (or recently quit <12 months) | 6 / 18 (33.3) |

*All data are self-reported

**Table S4: Baseline Demographics of participants who did not respond at 1 month**

| **Characteristic*** | **N = 110** |
| --- | --- |
| **Sex, n(%)** |  |
| Female | 23 / 110 (20.9) |
| **Age** | 60.2 (12.8) |
| (Missing) | 3 |
| **Ethnicity, n(%)** |  |
| Aboriginal/Torres Strait Islander | 2 / 110 (1.8) |
| Australian/New Zealand | 18 / 110 (16.4) |
| Polynesian | 1 / 110 (0.9) |
| European | 28 / 110 (25.5) |
| American (North, Central and South) | 1 / 110 (0.9) |
| South Asian (Bangladesh, India, Nepal, Pakistan, Sri Lanka) | 25 / 110 (22.7) |
| East Asian (China, Japan, Taiwan) | 8 / 110 (7.3) |
| South-East Asian (Vietnam, Cambodia, Laos, Burma, Malaysia, Singapore, Philippines, Thailand, Indonesia, East Timor) | 7 / 110 (6.4) |
| Middle-East and North African | 18 / 110 (16.4) |
| Sub-Saharan Africa | 0 / 110 (0) |
| Pacific Islander | 2 / 110 (1.8) |
| Other | 0 / 110 (0) |
| **Preferred Languages, n(%)** |  |
| Arabic | 9 / 110 (8.2) |
| Mandarin (Simplified Chinese subtitles) | 6 / 110 (5.5) |
| English | 82 / 110 (74.5) |
| Hindi | 13 / 110 (11.8) |
| **Education level, n(%)** |  |
| Never attended school | 1 / 110 (0.9) |
| Primary | 7 / 110 (6.4) |
| Secondary school without completion certificate | 23 / 110 (20.9) |
| Secondary school graduate | 15 / 110 (13.6) |
| Technical or Vocational qualifications | 11 /110 (10) |
| University undergraduate | 41 / 110 (37.3) |
| University postgraduate | 12 / 110 (10.9) |
| **Risk Factors, n(%)** |  |
| Diabetes | 43 / 110 (39.1) |
| Hypertension | 66 / 110 (60.0) |
| High Cholesterol | 58 / 110 (52.7) |
| Smoker (or recently quit <12 months) | 41 / 110 (37.3) |
| *All data is self-reported | |

## **Supplement I| Table S5- 16: Post hoc Analysis**

### **Table S5. Proportion of participants who improved their total number of correct responses from baseline to post-intervention**

|  | |
| --- | --- |
| **Characteristic** | **N = 129***^1*^* |
| **Proportion of participants who improved their total number of correct responses from baseline to post-intervention** | 93 / 128 (72.7%) |
| (Missing) | 1 |
| *^1^* n / N (%) | |

| **Table S6-S16: Exploratory Ten item level McNemar tests** **Table S6: 2x2 table for Question 1 (Q1) before and after the intervention** | | | | |  |
| --- | --- | --- | --- | --- | --- |
|  | **Number of Q1 responses correct post-intervention** | | | **Total** | **p-value*** |
|  | Did not get the response correct | Got the response correct | (Missing) |  |  |
| **Number of Q1 responses correct at baseline** |  |  |  |  | 0.025 |
| Did not get the response correct | 14 | 18 | 1 | 33 |  |
| Got the response correct | 6 | 90 | 0 | 96 |  |
|  |  |  |  |  |  |
| **Total** | 20 | 108 | 1 | 129 |  |
| *p-value comparing the average number of correct response post intervention vs pre-intervention (Q1)  Q1. A heart attack is most commonly caused by? blockage of a heart artery | | | | |  |

| **Table S7: 2x2 table for Question 2 (Q2) before and after the intervention** | | | | |  |
| --- | --- | --- | --- | --- | --- |
|  | **Number of Q2 responses correct post-intervention** | | | **Total** | **p-value*** |
|  | Did not get the response correct | Got the response correct | (Missing) |  |  |
| **Number of Q2 responses correct at baseline** |  |  |  |  | <0.001 |
| Did not get the response correct | 42 | 47 | 0 | 89 |  |
| Got the response correct | 1 | 38 | 1 | 40 |  |
|  |  |  |  |  |  |
| **Total** | 43 | 85 | 1 | 129 |  |
| * P-value comparing the average number of correct responses post intervention vs pre-intervention (Q2)  Q2. Blood thinning medications given after a myocardial infarction (heart attack) reduce blood clot by working on: Platelets | | | | | |

| **Table S8: 2x2 table for Question 3 (Q3) before and after the intervention** | | | | |  |
| --- | --- | --- | --- | --- | --- |
|  | **Number of Q3 responses correct post-intervention** | | | **Total** | **p-value*** |
|  | Did not get the response correct | Got the response correct | (Missing) |  |  |
| **Number of Q3 responses correct at baseline** |  |  |  |  | 0.039 |
| Did not get the response correct | 12 | 12 | 0 | 24 |  |
| Got the response correct | 3 | 101 | 1 | 105 |  |
|  |  |  |  |  |  |
| **Total** | 15 | 113 | 1 | 129 |  |
| * P-value comparing the average number of correct responses post intervention vs pre-intervention (Q3)  Q3. Aspirin is used after myocardial infarction as a: A blood thinner to stop blood clots | | | | | |

| **Table S9. 2x2 table for Question 4 (Q4) before and after the intervention** | | | | |  |
| --- | --- | --- | --- | --- | --- |
|  | **Number of Q4 responses correct post-intervention** | | | **Total** | **p-value*** |
|  | Did not get the response correct | Got the response correct | (Missing) |  |  |
| **Number of Q4 responses correct at a baseline** |  |  |  |  | <0.001 |
| Did not get the response correct | 11 | 20 | 0 | 31 |  |
| Got the response correct | 1 | 96 | 1 | 98 |  |
|  |  |  |  |  |  |
| **Total** | 12 | 116 | 1 | 129 |  |
| * P-value comparing the average number of correct responses post intervention vs pre-intervention (Q4)  Q4. After a heart attack aspirin is an essential medication which should be taken: Long term until instructed to stop by your doctor | | | | |  |

| **Table S10. 2x2 table for Question 5 (Q5) before and after the intervention** | | | | |  |
| --- | --- | --- | --- | --- | --- |
|  | **Number of Q5 responses correct post-intervention** | | | **Total** | **p-value*** |
|  | Did not get the response correct | Got the response correct | (Missing) |  |  |
| **Number of Q5 responses correct at a baseline** |  |  |  |  | <0.001 |
| Did not get the response correct | 29 | 31 | 0 | 60 |  |
| Got the response correct | 4 | 64 | 1 | 69 |  |
|  |  |  |  |  |  |
| **Total** | 33 | 95 | 1 | 129 |  |
| * P-value comparing the average number of correct responses post intervention vs pre-intervention (Q5)  Q5. Clopidogrel ('Plavix') and Ticagrelor ('Brilinta') are blood thinners used after a myocardial infarction which are given to: Stop blood clot forming on the stent within the heart artery | | | | |  |

| **Table S11. 2x2 table for Question 6 (Q6) before and after the intervention** | | | | |  |
| --- | --- | --- | --- | --- | --- |
|  | **Number of Q6 responses correct post-intervention** | | | **Total** | **p-value*** |
|  | Did not get the response correct | Got the response correct | (Missing) |  |  |
| **Number of Q6 responses correct at a baseline** |  |  |  |  | <0.001 |
| Did not get the response correct | 43 | 36 | 1 | 80 |  |
| Got the response correct | 9 | 40 | 0 | 49 |  |
|  |  |  |  |  |  |
| **Total** | 52 | 76 | 1 | 129 |  |
| * P-value comparing the average number of correct responses post intervention vs pre-intervention (Q6)  Q6. After a heart attack the most effective way of preventing future heart attacks is to: Treatment with cholesterol lowering medications | | | | | |

| **Table S12. 2x2 table for Question 7 (Q7) before and after the intervention** | | | | |  |
| --- | --- | --- | --- | --- | --- |
|  | **Number of Q7 responses correct post-intervention** | | | **Total** | **p-value*** |
|  | Did not get the response correct | Got the response correct | (Missing) |  |  |
| **Number of Q7 responses correct at a baseline** |  |  |  |  | 0.264 |
| Did not get the response correct | 14 | 13 | 0 | 27 |  |
| Got the response correct | 7 | 94 | 1 | 102 |  |
|  |  |  |  |  |  |
| **Total** | 21 | 107 | 1 | 129 |  |
| * P-value comparing the average number of correct responses post intervention vs pre-intervention (Q7)  Q7. Narrowing of the coronary (heart) arteries is usually caused by: A build up of fat in the heart artery wall | | | | | |

| **Table S13. 2x2 table for Question 8 (Q8) before and after the intervention** | | | | |  |
| --- | --- | --- | --- | --- | --- |
|  | **Number of Q8 responses correct post-intervention** | | | **Total** | **p-value*** |
|  | Did not get the response correct | Got the response correct | (Missing) |  |  |
| **Number of Q8 responses correct at a baseline** |  |  |  |  | <0.001 |
| Did not get the response correct | 57 | 26 | 0 | 83 |  |
| Got the response correct | 6 | 39 | 1 | 46 |  |
|  |  |  |  |  |  |
| **Total** | 63 | 65 | 1 | 129 |  |
| * P-value comparing the average number of correct responses post intervention vs pre-intervention (Q8)  Q8. Cholesterol lowering medications are prescribed after a myocardial infarction: Regardless of your cholesterol level | | | | | |

| **Table S14. 2x2 table for Question 9 (Q9) before and after the intervention** | | | | |  |
| --- | --- | --- | --- | --- | --- |
|  | **Number of Q9 responses correct post-intervention** | | | **Total** | **p-value*** |
|  | Did not get the response correct | Got the response correct | (Missing) |  |  |
| **Number of Q9 responses correct at a baseline** |  |  |  |  | <0.001 |
| Did not get the response correct | 41 | 45 | 0 | 86 |  |
| Got the response correct | 4 | 38 | 1 | 43 |  |
|  |  |  |  |  |  |
| **Total** | 45 | 83 | 1 | 129 |  |
| * P-value comparing the average number of correct responses post intervention vs pre-intervention (Q9)  Q9. Medications such as ACE inhibitors (ending in 'pril') or ARBs (ending in 'sartan') reduce the amount of work that the heart has to do by: reducing the blood pressure | | | | | |

| **Table S15. 2x2 table for Question 10 (Q10) before and after the intervention** | | | | |  |
| --- | --- | --- | --- | --- | --- |
|  | **Number of Q10 responses correct post-intervention** | | | **Total** | **p-value*** |
|  | Did not get the response correct | Got the response correct | (Missing) |  |  |
| **Number of Q10 responses correct at a baseline** |  |  |  |  | <0.001 |
| Did not get the response correct | 44 | 39 | 0 | 83 |  |
| Got the response correct | 5 | 40 | 1 | 46 |  |
|  |  |  |  |  |  |
| **Total** | 49 | 79 | 1 | 129 |  |
| * P-value comparing the average number of correct responses post intervention vs pre-intervention (Q10)  Q10. Beta blockers reduce stress on the heart by: reducing the heart rate | | | | |  |

### **Table S16: Proportion of participants who met medication knowledge target (correctly answering all 7 medication questions)** **comparing pre-intervention to post-intervention baseline vs post-intervention**

|  | Baseline (N=129) | Immediately post-intervention (N=128) | p-value |
| --- | --- | --- | --- |
| Proportion of participants who met medication knowledge target * | 9/129 (7.0%) | 29 / 128 (22.7%) | < 0.001 |
| * Analysed with Exploratory McNemar’s test. |  |  |  |

## **Supplement J| Table S17. Acceptability measures immediately post-intervention and at 1 month follow-up**

| Characteristic | post-intervention,  n / N (%)  N = 128 | 1 month,  n / N (%)  N = 18 |
| --- | --- | --- |
| Q1. The information delivered in the video was easy enough to understand |  |  |
| Strongly disagree (1) | 1 / 128 (0.8%) | 0 / 18 (0.0%) |
| Disagree (2) | 5 / 128 (3.9%) | 0 / 18 (0.0%) |
| Neutral (3) | 15 / 128 (11.7%) | 1 / 18 (5.6%) |
| Agree (4) | 52 / 128 (40.6%) | 5 / 18 (27.8%) |
| Strongly Agree (5) | 55 / 128 (43.0%) | 12 / 18 (66.7%) |
| Q2. I found the information useful |  |  |
| Strongly disagree (1) | 2 / 128 (1.6%) | 0 / 18 (0.0%) |
| Disagree (2) | 0 / 18 (0.0%) | 0 / 18 (0.0%) |
| Neutral (3) | 14 / 128 (10.9%) | 1 / 18 (5.6%) |
| Agree (4) | 54 / 128 (42.2%) | 4 / 18 (22.2%) |
| Strongly Agree (5) | 58 / 128 (45.3%) | 13 / 18 (72.2%) |
| Q3. I found the video engaging |  |  |
| Strongly disagree (1) | 2 / 128 (1.6%) | 0 / 18 (0.0%) |
| Disagree (2) | 4 / 128 (3.1%) | 0 / 18 (0.0%) |
| Neutral (3) | 27 / 128 (21.1%) | 1 / 18 (5.6%) |
| Agree (4) | 50 / 128 (39.1%) | 6 / 18 (33.3%) |
| Strongly Agree (5) | 45 / 128 (35.2%) | 11 / 18 (61.1%) |
| Q4. Would you like to receive similar videos in the future? |  |  |
| Yes | 108 / 128 (84.4%) | 16 / 18 (88.9%) |
| No | 20 / 128 (15.6%) | 2 / 18 (11.1%) |
