# Supplementary material for: Multilingual Video Education for Hospitalized Patients With Myocardial Infarction (EDUCATE-MI): Single-Arm Implementation Study
Source: JMIR Cardio. 2026 Mar 26;10:e82817. doi: 10.2196/82817 (PMC13020905; doi:10.2196/82817)
Supplement: Checklist 2 [file cardio-v10-e82817-s003.docx]

## **Supplement B| eMethods 2: TIDier Checklist**

**
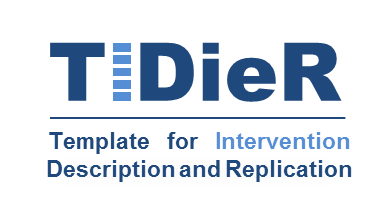
The TIDieR (Template for Intervention Description and Replication) Checklist*:**

Information to include when describing an intervention and the location of the information

| **Item number** | **Item** | **Where located **** | |
| --- | --- | --- | --- |
|  |  | Primary paper  (page or **Supplement**  number) | Other ^†^ (details) |
|  | **BRIEF NAME** | Title page |  |
| **1.** | EDUCATE MI | ____________ | ______________ |
|  | **WHY** | Supplement B\| eMethods 2: TIDier Checklist |  |
| **2.** | Goal of the intervention was to address an implementation gap of a need to provide patient education to patients post MI in a tertiary hospital with diverse multi-cultural population. A potential solution was to create a video that could be adapted to three most commonly spoken languages to bridge this gap. | ____________ | _____________ |
|  | **WHAT** | Supplement B\| eMethods 2: TIDier Checklist |  |
| **3.** | Materials: Patient education on myocardial infarction (MI; heart attack), its causes and management (with a focus on post-MI medications) was delivered in a 5-minute video. This video can be accessed via a URL to Vimeo. | ____________ | _____________ |
| **4.** | Procedures: The intervention (i.e. a video) was designed by investigators (AT and SK). Video development required a minimum time commitment of four hours with additional time required to create new content like animations and figures to complement the narration. In brief, after consultation with the latest acute coronary syndrome guidelines, investigators developed a transcript in plain English that addressed the fundamental concepts of MI pathophysiology, and management (particularly focussing on medications). Videos sequence with animations were created with Video Scribe video animation software version 3.6 (Sparkol) at a cost ($AUD42 per month). A voiceover of the transcript was recorded by SK, and we ensured the voiceover corresponded to the video sequence. During this time, the survey questionnaires were developed. Readability score of the English video transcript was Grade 10 (see Figure S1) and survey questionnaires were Grade 9. We tailored the video to other languages by adjusting the video sequence to match the content of the spoken language. We collected verbal feedback from participants on the non-English videos to collect data for future intervention refinement. | ____________  Supplement B\| eMethods 2: TIDier Checklist | _____________ |
|  | **WHO PROVIDED** | Page 6  Supplement B\| eMethods 2: TIDier Checklist |  |
| **5.** | Research assistant (AZ) provided the video to the participant via a tablet (e.g. iPad). Participants also had the option to scan a QR code to access the intervention. Research assistant was present when the participant was watching the video. | ____________ | _____________ |
|  | **HOW** | Page 6  Supplement B\| eMethods 2: TIDier Checklist |  |
| **6.** | The video was delivered face-to-face individually to patients via a tablet. | ____________ | _____________ |
|  | **WHERE** |  |  |
| **7.** | The video was delivered in cardiology ward at a tertiary hospital in Sydney, Australia. | _____________ | _____________ |
|  | **WHEN and HOW MUCH** | Page 6  Supplement B\| eMethods 2: TIDier Checklist |  |
| **8.** | The video was delivered once at the time of recruitment and is accessible on a survey webpage after the participant completes the baseline surveys. Duration of the video was 5 minutes and participants had an option to receive a link for 1-month follow-up containing the video. | _____________ | _____________ |
|  | **TAILORING** | Supplement B\| eMethods 2: TIDier Checklist |  |
| **9.** | The video audio and subtitles were adapted to three other languages: Arabic, Hindi and Mandarin.  These languages were selected because they are the three most commonly spoken Non-English languages in the local health district.^1^ The process of translation was performed after the English video was finalised, following the Australian Institute of Interpreters and Translators’ Guidelines.^2^ The transcripts for the video audio and captions for subtitles, patient information and consent form and survey landing pages were translated and back translated by accredited translators from Western Sydney Local Health District (WSLHD) Translation Service. These healthcare translators were accredited by the National Accreditation Authority for Translators and Interpreters (NAATI). All translations were also reviewed by native speakers of each language and an independent translator and discrepancies were arbitrated through discussion. Voiceovers were performed by bilingual speakers who were clinicians. Videos were pre-tested with a convenience sample of consumers who were native speakers of the different preferred languages.  Furthermore, readability score of the English video transcript and questionnaires was obtained from a readability calculator. ^3^ | _____________ | _____________ |
|  | **MODIFICATIONS** | N/A |  |
| **10.^ǂ^** | N/A | _____________ | _____________ |
|  | **HOW WELL** | N/A |  |
| **11.** | Planned: 4 videos available to be delivered | _____________  N/A | _____________ |
| **12.^ǂ^** | Actual: No modifications to videos- videos were delivered as planned | _____________ | _____________ |

** **Authors** - use N/A if an item is not applicable for the intervention being described. **Reviewers** – use ‘?’ if information about the element is not reported/not sufficiently reported.

† If the information is not provided in the primary paper, give details of where this information is available. This may include locations such as a published protocol or other published papers (provide citation details) or a website (provide the URL).

ǂ If completing the TIDieR checklist for a protocol, these items are not relevant to the protocol and cannot be described until the study is complete.

* We strongly recommend using this checklist in conjunction with the TIDieR guide (see *BMJ* 2014;348:g1687) which contains an explanation and elaboration for each item.

* The focus of TIDieR is on reporting details of the intervention elements (and where relevant, comparison elements) of a study. Other elements and methodological features of studies are covered by other reporting statements and checklists and have not been duplicated as part of the TIDieR checklist. When a **randomised trial** is being reported, the TIDieR checklist should be used in conjunction with the CONSORT statement (see [www.consort-statement.org](http://www.consort-statement.org)) as an extension of **Item 5 of the CONSORT 2010 Statement.** When a **clinical trial** **protocol** is being reported, the TIDieR checklist should be used in conjunction with the SPIRIT statement as an extension of **Item 11 of the SPIRIT 2013 Statement** (see [www.spirit-statement.org](http://www.spirit-statement.org)). For alternate study designs, TIDieR can be used in conjunction with the appropriate checklist for that study design (see [www.equator-network.org](http://www.equator-network.org)).

**
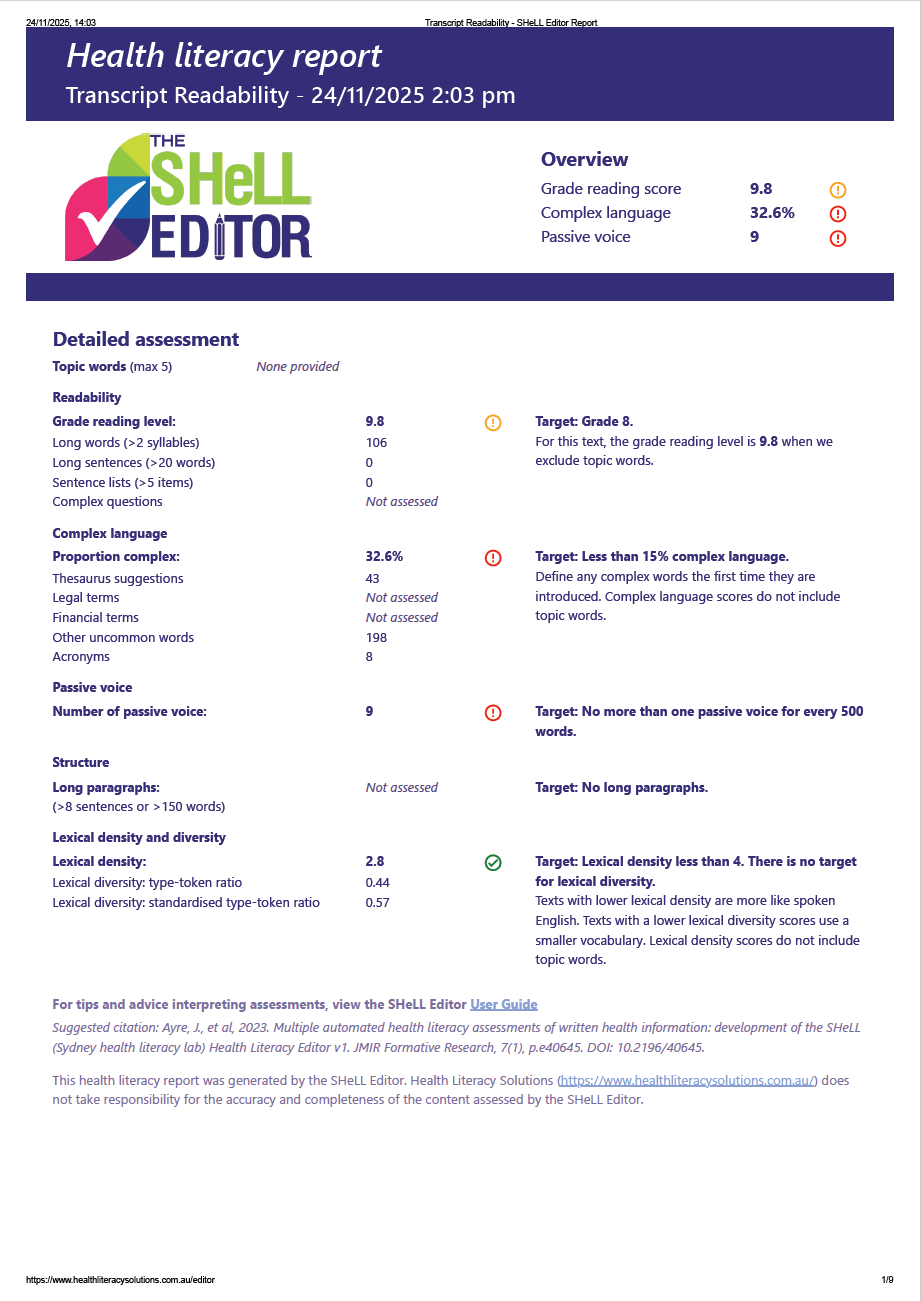
** **
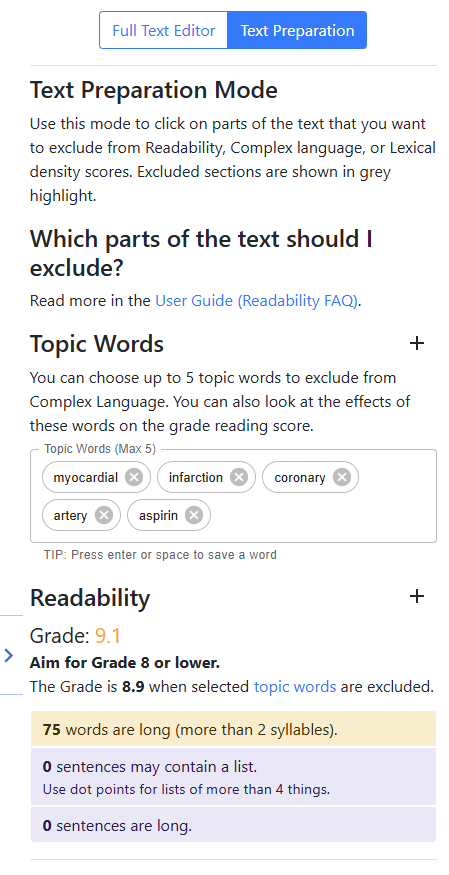
**

A

B

**Figure S1: Summary reports of the Readability Score of the Video Transcript (English);** A: Report with Original Transcript; B: List of complex words excluded and the corresponding readability score

**References**

1. Strategic Priorities - WSLHD. Accessed March 17, 2025. https://www.wslhd.health.nsw.gov.au/Better-West/Strategic-Priorities

2. Hlavac J. The Development of Community Translation and Interpreting in Australia: A Critical Overview. In: *Translating and Interpreting in Australia and New Zealand*. Routledge; 2021.

3. Ayre J, Bonner C, Muscat DM, et al. Multiple Automated Health Literacy Assessments of Written Health Information: Development of the SHeLL (Sydney Health Literacy Lab) Health Literacy Editor v1. *JMIR Form Res*. 2023;7:e40645. doi:10.2196/40645
