# Supplementary material for: Multilingual Video Education for Hospitalized Patients With Myocardial Infarction (EDUCATE-MI): Single-Arm Implementation Study
Source: JMIR Cardio. 2026 Mar 26;10:e82817. doi: 10.2196/82817 (PMC13020905; doi:10.2196/82817)
Supplement: Checklist 4 [file cardio-v10-e82817-s005.docx]

## **Supplement K| Table S18: iCHECK-DH: Guidelines and Checklist for the Reporting on Digital Health Implementations**

| Section |  | Item | Description | Page it’s reported |
| --- | --- | --- | --- | --- |
| Title | 1 | Title  (M1) | Identification as an implementation report, and description of the implementation  in the title and/or keywords | 0 |
| Abstract | 2 | Abstract  (M) | Provide a summary of the key elements of the implementation report, including a  description of the implementation strategy, the intervention, defining the key  elements of the implementation and health outcomes and specify the key  KPIs/Outputs. We recommend describing the main aspects of the research in the  following order: Background - Objectives - Methods - Implementation (Results) -  Conclusions - (Optional: Trial Registration). | 1 |
| Introduction | 3 | Context  (M) | Describe the geographical areas, organizations, target populations and  implementation context. Consider social, cultural, economic, political, health care  and organizational barriers, infrastructures and facilitators that may influence  implementation elsewhere. Explicitly highlight whether a national digital health  strategy exists and whether implementation is aligned with the strategy.  Describe the stage of the implementation (Developing or Adapting Solution /  Piloting and Evidence generation / Package and Advocacy /Acceleration / Deploying  / Scaling up / Hand over or Complete).2 | 3 |
|  | 4 | Problem  statement  (M) | Description of the health care or public health problem, challenge, or deficiency that  the implementation aims to address. (If applicable, include a reference to the 'health  system challenge' of the WHO Classification of Digital Health Interventions3 in the  description) | 3 |
|  | 5 | Similar  Interventions  (M) | Mention whether this implementation was inspired by another existing one, and if  so, what is the added value of your intervention, if any, compared to the initial one?  And what, if anything, has been done differently? | NA |
| Methods | 6 | Aims and  Objectives  (M) | Describe the main objectives and the overall aim of the implementation. Describe  how these will be measured using predefined primary and secondary outcome(s)  and key performance indicators for this implementation and the expected  intervention(s).  For example: indicators or proxy-indicators measuring direct health outcomes (e.g.,  HbA1c for diabetic patients); Key Performance Indicators (e.g., number of users,  number of users that are properly trained, user satisfaction); Indicator assessing a  particular process (e.g., administrative time for patient admission);  (If there was no evaluation, provide detailed explanation for reasoning) | 4 |
|  | 7 | Blueprint  summary  (M) | Describe the design and key features of the intervention and key points of the  implementation strategy and roadmap. | 4 |
|  | 8 | Technical Design  (M) | Reasons for developing or choosing this tool. Does it combine several tools? Provide  a brief description of the tool(s) (functionality and architecture) and how it fits into  the health enterprise architecture and investment roadmap (if applicable). Indicate  whether the solution is based on an existing solution or has been developed or  purchased specifically for this intervention.  Describe the type of technology used (e.g., AI applications), license of the  technology (open source, free, commercial, IP ownership etc.), include code  documentation (if available), link to the application, link to wiki or project website. | 4 |
|  | 9 | Target  (M) | The target refers to the focus or recipient of the intervention. It is the specific  person, group, system, or problem that the intervention aims to change or improve.  The characteristics of the targeted "site(s)" (locations, staff, resources, etc.) for  implementation and any eligibility criteria. The population targeted by the  intervention and any eligibility criteria. | 4 |
|  | 10 | Data  (M) | Describe the data governance, including life cycle (collection, processing, storage,  modification, sharing, suppression), the data ownership (mention whether patients  actually have access to the data), data protection measures, confidential use of  routine data, expected level of data integration, data for research, cross-border  data agreement, if any, the applicable legal framework, and how the project  complies with it. Data consent: Has patient consent been obtained? Describe the  approach to data protection and cybersecurity (e.g. security by design, privacy by  design, etc.) and where the data is hosted. (e.g., in-country, cloud based, hybrid  model etc.). Describe, if applicable, the government preferences in terms of data  policies. | N/A |
|  | 11 | Interoperability  (M) | Describe the interfaces (what other systems does the tool connect to) and the  standards that were used (which specific ones and rationale of choice) (e.g.,  semantic ontologies such ICD as SNOMED, LOINC or technical standards such as HL7  FHIR, etc.). | 4-5 |
|  | 12 | Participating  entities  (M) | Describe the implementing organization(s): Type of organisation(s), mission,  leadership, vision, etc.  Government involvement: Describe whether the government was involved in the  implementation, at what level and at what stage(s).  Partners: Describe all partners (organisations) and their role in the implementation.  Funders: List all actors and stakeholders who have funded or invested in the  development of the implementation (if different from the implementation, e.g.  using an existing digital health intervention). Indicate their level of involvement in  terms of funding.  Mention which entity will own the final product and intellectual property after the  implementation phase. | Supplement D |
|  | 13 | Budget Planning  (M) | Describe the planned budget for implementation (include costs such as change  management, user training, project management, technology pricing, total cost of  ownership). If possible, include actual costs, otherwise describe the range or  percentage of the total budget. Indicate the period covered by the budget. Describe  the budget for the intervention (e.g. development, purchase or adaptation of a free  tool); if possible include real costs, otherwise describe as a percentage of the total  budget. Indicate the duration covered by the budget. | Not reported |
|  | 14 | Sustainability  (M) | Describe the Business model including the sustainability model (financial, economic,  environment etc.). If possible, put outcomes in relation to cost to assess  sustainability. Describe long term exit strategies, and all dimensions considered to  sustain the project after the end of funding. If applicable, describe potential  institutionalization of the project. | Not reported |
| Results | 15 | Coverage  (M) | Describe whether the coverage of implementation is international, national,  regional or at the level of e.g. municipalities. If coverage is sub-national, describe  the regions. Provide information on the relative importance of the coverage (e.g. %  of eligible population covered). | 4 |
|  | 16 | Outcomes  (M) | Primary and other outcome(s) of the implementation. Detail the actual outcomes,  using the pre-defined outcome measures (if applicable). | 5 |
|  | 17 | Lessons learned  (M) | Describe any lessons learned from the implementation experience that could be  used to improve future outcomes. This could include, but is not limited to, success  factors, implementation challenges or budget considerations.  Success factors: Describe factors that positively influenced the implementation (e.g.  involvement of key stakeholders). Also describe contextual factors that may have  positively influenced the results (e.g. new legal requirements that facilitated  adoption).  Challenges to implementation: Describe challenges (process-related, such as  resistance to change, but also technical). Include contextual factors that may have  affected the achievement of outcomes (e.g. unexpected change of government).  Budget: Describe whether the implementation budget was adhered to, and if not,  why not. Also detail the expected operational costs (e.g. license, maintenance,  human resources, updates to in-house developments) to estimate the total cost of  ownership. Include real costs, otherwise describe them as a percentage of the total  budget.  What recommendations can be drawn from the lessons learned? | 9-11 |
|  | 18 | Unintended  consequences  (NM4) | Describe unintended consequences (positive or negative), harms or negative side effects  (if any). | Not reported |
| Discussion | 19 | Conclusion  (M) | Summary of the conclusions and future implications. | 15-18 |
| General | 20 | General  (NM) | If applicable, include statement(s) on regulatory approvals (including, as  appropriate, ethical approval, governance approval), trial or study registration  (availability of protocol), and conflicts of interest. | 8 |
